# A matter arising: When should inflammatory and autoimmune rheumatic diseases be considered ‘early’?

**DOI:** 10.1111/eci.70136

**Published:** 2025-10-21

**Authors:** Elvis Hysa, Emanuele Gotelli, Carmen Pizzorni, Sabrina Paolino, Alberto Sulli, Vanessa Smith, Rosanna Campitiello, Maurizio Cutolo

**Affiliations:** ^1^ Laboratory of Experimental Rheumatology and Academic Division of Clinical Rheumatology, Department of Internal Medicine (DiMI) University of Genova Genoa Italy; ^2^ Department of Experimental Medicine (DIMES) University of Genova Genoa Italy; ^3^ IRCSS Ospedale Policlinico San Martino Genoa Italy; ^4^ Department of Internal Medicine Ghent University Ghent Belgium; ^5^ Department of Rheumatology Ghent University Hospital Ghent Belgium; ^6^ Unit for Molecular Immunology and Inflammation VIB Inflammation Research Center (IRC) Ghent Belgium

**Keywords:** autoimmune rheumatic diseases, biomarkers, early diagnosis, imaging, inflammation

## Abstract

**Background:**

Early diagnosis is pivotal for guiding the intensity of clinical monitoring, optimizing therapeutic strategies and preventing organ damage in inflammatory and autoimmune rheumatic diseases (IARDs). This review summarizes current evidence on early diagnostic and therapeutic approaches of some IARDs, including rheumatoid arthritis (RA), systemic sclerosis (SSc) and detection of large‐vessel vasculitis (LVV) in polymyalgia rheumatica (PMR), representing distinct pathophysiological mechanisms of joint synovitis, tissue fibrosis and vasculitis, respectively.

**Methods:**

A comprehensive narrative literature review was conducted focusing on early recognition strategies, searching PubMed and Scopus databases with emphasis on studies from the past 5 years and recent EULAR/ACR conference abstracts (2023–2025).

**Results:**

In RA, clinically suspect arthralgia with seropositivity for rheumatoid factor and anti‐citrullinated peptide antibodies significantly increases progression risk to definite RA. Musculoskeletal ultrasound detects subclinical synovitis in 44%–51% of high‐risk individuals, while MRI identifies bone marrow edema predicting erosive progression. Abatacept significantly reduces RA development in seropositive individuals at high risk of RA.

In SSc, Raynaud's phenomenon combined with SSc‐specific autoantibodies and abnormal nailfold capillaroscopy predicts progression to definite disease, with 79.5% developing SSc within 4.6 years. LeRoy's criteria, validated by Koenig, enables early identification, though evidence for disease‐modifying interventions in preclinical stages remains limited.

For PMR, imaging reveals subclinical LVV in 16%–23% of patients without cranial symptoms. Subclinical LVV associates with higher relapse rates in retrospective studies, though optimal management approaches require prospective validation.

**Conclusions:**

Advances in early IARD recognition through refined clinical criteria, enhanced biomarkers and imaging enable risk stratification and personalized management. While intervention strategies show promise, particularly in RA, optimal patient selection and treatment protocols require further research.

## Graphical abstract

Timely recognition of inflammatory and autoimmune rheumatic diseases (IARDs) is important to optimize the early diagnosis with tailored interventions and possible prevention of irreversible organ damage. This narrative review provides an update by summarizing the advances in identifying the early stages of rheumatoid arthritis, systemic sclerosis and large‐vessel vasculitis in polymyalgia rheumatica, representing distinct mechanisms of synovitis, fibrosis and vasculitis. Combining clinical, serological and imaging tools enhances early detection of risk factors for progression.

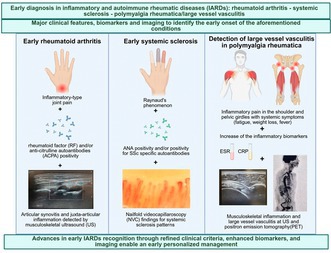

## 1 INTRODUCTION

Early, and when possible, very early, diagnosis of inflammatory and autoimmune rheumatic diseases (IARDs) is crucial to minimize the impact of recognized trigger factors and to allow for timely, appropriate management strategies. These may include close monitoring and/or early therapeutic intervention, with the goal of preventing progressive and irreversible organ damage.

Healthcare professionals involved in the management of these conditions should be familiar with the initial clinical manifestations, as well as laboratory markers and imaging findings, which can support prompt therapeutic decisions or closer monitoring to anticipate the evolution toward overt disease.

In this narrative review, we explore the current understanding of very early/early diagnosis of rheumatoid arthritis (RA), systemic sclerosis (SSc) and large vessel vasculitis (LVV) in patients with polymyalgia rheumatica (PMR).

These conditions were selected to represent inflammatory and ARDs with distinct pathophysiological mechanisms, namely synovial inflammation, tissue fibrosis, and vasculitis, respectively. This selection is also informed by the authors' clinical and research expertise in these specific areas. While we acknowledge that other important IARDs, such as systemic lupus erythematosus (SLE) and psoriatic arthritis (PsA), also have well‐characterized preclinical phases, involving the sequential development of autoantibodies or the transition from cutaneous psoriasis to joint inflammation, respectively^1^, ^2^, ^3^ our focused approach allows for an in‐depth discussion of these three core pathological processes.

A thorough narrative literature overview is provided with a major focus on the most recent findings, particularly studies published over the past 5 years, to highlight advances in early recognition and intervention strategies across these diverse conditions.

## 2 METHODS

This narrative review synthesizes the current evidence on early diagnostic strategies for RA, SSc, and LVV in patients with PMR. A comprehensive search of PubMed and Scopus databases was performed using relevant keywords, including ‘early diagnosis’, ‘rheumatoid arthritis”, ‘systemic sclerosis’, “large vessel vasculitis’, ‘polymyalgia rheumatica’, ‘biomarkers’ and ‘imaging’. While no date restrictions were applied to the database search to ensure an extensive review of the literature, particular emphasis was placed on studies published within the past 5 years to highlight recent advances. Additionally, abstracts presented at the annual congresses of the European Alliance of Associations for Rheumatology (EULAR) and the American College of Rheumatology (ACR) from 2023 to 2025 were screened to capture the most recent findings not yet published as full‐text manuscripts.

Reference lists of included studies were also examined to identify additional relevant publications that may not have appeared in the initial database search. All relevant articles and abstracts were critically appraised, and their contributions to understanding early diagnostic approaches across these distinct diseases were qualitatively synthesized.

The selection process prioritized studies providing high‐quality evidence and significant contributions to the field of early diagnosis and intervention across IARDs. Our inclusion principles favored systematic reviews, meta‐analyses, randomized controlled trials, large population‐based or registry studies, recent national or international guidelines and consensus statements. All relevant articles and abstracts were critically appraised by the authors based on their methodological rigor, relevance to the review's scope and the novelty and impact of their findings. The contributions of each study to the understanding of early diagnostic and therapeutic approaches across these distinct diseases were then qualitatively synthesized.

For interventional studies, a qualitative appraisal of the level of evidence was performed to guide the critical synthesis. This appraisal was based on different factors: the study design, with a strong preference for randomized controlled trials (RCTs); the study's primary outcome, where trials successfully meeting their primary endpoint with a sustained clinical benefit were considered to provide a high level of evidence; and the nature and durability of the effect. Accordingly, RCTs that failed to meet their primary endpoint, demonstrated only a temporary benefit, or were stopped early for futility were graded as providing moderate or low‐level evidence. This structured appraisal was used to contextualize the findings within our manuscript. Although not representing a formal systematic evidence‐grading score, this structured qualitative appraisal aligns with contemporary standards for high‐quality narrative reviews, which emphasize transparent and justified literature selection.^4^

### 2.1 Definitions of ‘early’ disease across the included IARDs

To provide a clear framework, the concept of ‘early disease’ is defined for each condition from both a pathophysiological ground and clinical perspectives.

For RA, the pathophysiological onset begins with detectable immune system dysregulation (seropositivity for rheumatoid factor and/or anti‐citrullinated peptide autoantibodies) and/or subclinical synovitis by imaging, potentially years before clinical symptoms.^5^ Clinical onset refers to the interval from the initial inflammatory joint symptoms up to fulfilment of criteria such as the 2010 ACR/EULAR,^6^ commonly within 3–12 months of onset, which is considered a critical window for intervention.

For SSc, the pathophysiological phase includes microvascular dysfunction, often presenting as Raynaud's phenomenon (RP) with evidence of a scleroderma‐pattern at nailfold capillaroscopy (NVC) and SSc‐specific serology, prior to skin or organ involvement, as described by the LeRoy/Medsger.^7^ Clinically, ‘early SSc’ applies to patients meeting these criteria before skin thickening or major organ involvement.

For patients with LVV manifesting with PMR symptoms, the concept of an early phase differs from the progressive models seen in RA and SSc. Unlike those conditions, PMR is not considered a preclinical stage that evolves into GCA; rather, both are viewed as concurrent manifestations of a single disease spectrum.^8^ Therefore, ‘early detection’ in this context refers to identifying co‐existing, subclinical vasculitis in a patient at or near the time of their PMR diagnosis.^9^ The pathophysiological onset is the phase where this vessel inflammation is present but clinically silent, detectable only by imaging.^10^ These patients, now recognized for their higher risk, represent a group potentially in need of tailored management.

## 3 RHEUMATOID ARTHRITIS

Rheumatoid arthritis (RA) is a chronic autoimmune disease causing persistent joint inflammation, damage, and disability.^11^ It affects women two to three times more than men when the disease emerges between the third and fifth decades of life.^12^, ^13^, ^14^, ^15^ Early detection and treatment, even before clinical arthritis appears, are crucial to prevent joint damage.^16^ In this context, a treat‐to‐target approach aiming for remission or low disease activity helps slow disease progression and reduce long‐term damage.^17^

While the developmental pathway can vary, RA is often understood to progress through distinct phases, particularly in seropositive individuals. This classic model begins with isolated autoantibody positivity (seropositivity) for rheumatoid factor (RF) or often much earlier anti‐citrullinated peptide antibodies (ACPA), followed by a transition to inflammatory‐type joint pain without visible synovitis (clinically suspect arthralgia) and finally the onset of clinically evident arthritis.^16^ In contrast, the preclinical cascade in ‘seronegative’ RA may not follow the same pattern and is thought to involve an early increase in pro‐inflammatory cytokines like TNF‐α and IL‐6.^18^ This phase of inflammatory joint pain without clinical synovitis is often termed ‘clinically suspect arthralgia’. The EULAR definition of ‘arthralgia suspicious for progression to RA’, published in 2017, provides a specific framework for identifying high‐risk individuals.^19^ This definition includes several clinical features that increase the risk of progression, such as: recent symptom onset, involvement of metacarpophalangeal (MCP) joints, morning stiffness lasting at least 60 min, a predominance of symptoms in the morning, difficulty making a fist and a positive MCP squeeze test. The presence of a family history of RA or RA‐specific autoantibodies further heightens this risk.^19^, ^20^

When clinical synovitis appears, the threshold for morning stiffness duration decreases to 30 min. The Early Rheumatoid Arthritis (ERA) criteria, requiring at least three of five items: morning stiffness ≥30 min, arthritis in ≥3 joint areas, hand joint arthritis, positive RF, positive ACPA and improve early diagnosis sensitivity (84.4% vs. 58% of older criteria), enabling timely treatment.^21^, ^22^

### 3.1 Articular, systemic and extra‐articular symptoms of early rheumatoid arthritis

Joint pain is typically the first symptom reported by patients with early RA. Recent data suggest that individuals diagnosed within 3 months of symptom onset experience higher pain intensity compared to those with longer diagnostic delays. This association is likely to reflect the fact that more severe pain and functional limitations prompt patients to seek medical care more promptly, leading to an earlier diagnosis^23^ (see Table 1).

**TABLE 1:** Clinical and laboratory elements for the early diagnosis of RA and imaging features for the assessment of early joint involvement and damage.

	Finding	Main notes
Elements for early diagnosis of RA		
Clinical features	Joint pain	A primary symptom; higher intensity scores are reported in patients diagnosed within 3 months of onset.^23^
	Morning stiffness	Lasts ≥60 min in the clinically suspect arthralgia phase^24^
	Joint swelling	Present in ~75% of cases at initial presentation, typically affecting the small joints of the hands.^25^
	Systemic symptoms	Fatigue is a disabling early symptom, reported as severe by 59% of patients at baseline.^26^ Night pain is also common.^27^
	Early extra‐articular manifestations	Most frequent include sicca symptoms (~28%) and Raynaud's phenomenon (~17%). Rheumatoid nodules can occur early in 7%–23% of patients.^28^
Laboratory markers	Autoantibodies	Rheumatoid Factor (RF): Present in 50%–80% of patients^29^ ACPA: Present in 60%–80% of patients, with higher specificity (95%–98%)^29^ Anti‐CarP antibodies: Found in 20%–40%, but mainly used in research settings^29^
	Inflammatory markers	ESR and CRP are elevated at diagnosis in approximately 33%–56% of patients, but can be normal in up to 40% of cases^30^, ^31^
Assessment for early joint involvement and damage		
Imaging: musculoskeletal ultrasound (US)	Subclinical inflammation	Detects subclinical synovitis in 44%–51% of seropositive individuals with arthralgia.^32^ A Power Doppler signal is seen in ~30% of high‐risk cohorts.^33^ Tenosynovitis: an important early indicator, detected in 63%–85% of early RA patients.^34^
Imaging: magnetic resonance imaging (MRI)	Bone marrow edema (BME)	A key predictor of erosive progression, found in 41%–68% of patients in hand and wrist joints^35^
	Synovitis and tenosynovitis	MRI is highly sensitive, detecting synovitis in 83%–91% and tenosynovitis in 75%–85% of early RA patients^36^
	Early erosions	Can be found in 47%–72% of early RA patients with comprehensive multi‐joint MRI protocols^35^

Abbreviations: ACPA, anti‐citrullinated peptide antibodies; anti‐CarP, anti‐carbamylated protein antibodies; BME, bone marrow edema; CRP, C‐reactive protein; ESR, erythrocyte sedimentation rate; MRI, magnetic resonance imaging; RA, rheumatoid arthritis; RF, rheumatoid factor; US, ultrasound.

Symmetrical joint swelling, especially involving the small joints of the hands, is present in approximately 75% of cases, although about 25% present with an asymmetrical pattern, which can complicate early recognition.^23^, ^25^

Night pain and sleep disturbances, linked to nocturnal inflammation and altered circadian rhythms, are also frequently reported in the early phase of RA.^37^

Among the systemic features, fatigue is one of the most disabling symptoms in early RA, reported as severe by 59% and moderate by 19% of patients at baseline.^38^ In about 25% of cases, it persists over 5 years despite good disease control.^26^ Indeed, improvement in fatigue often lags behind clinical remission by up to 6 months, suggesting mechanisms beyond synovial inflammation.^38^

Extra‐articular manifestations (ExRA) can be a key feature of early RA, affecting 23%–41% of patients at or near diagnosis.^39^

The most frequent early ExRA include sicca symptoms (28%), RP (17%) and rheumatoid nodules, which can affect up to 23% of patients, particularly seropositive smokers.^28^, ^39^, ^40^ While more severe manifestations can occur, they are less common at initial presentation; for instance, subclinical interstitial lung disease (ILD) may be detectable and rheumatoid vasculitis is rare (<1%) in early disease.^28^, ^39^, ^41^

### 3.2 Laboratory markers

Anti‐citrullinated peptide antibodies (ACPA) and RF are the most established serological biomarkers for early RA. ACPAs offer greater specificity (90%–96%) compared to RF (70%–80% sensitivity but lower specificity), and both can precede clinical onset by several years (up to 15 years).^42^

Their combined presence markedly increases the risk of progression, particularly in individuals carrying the HLA‐DRB1 shared epitope. This genetic motif, located within the major histocompatibility complex (MHC) class II region, enhances antigen presentation of citrullinated peptides to autoreactive T cells and represents the strongest known genetic risk factor for RA development.^43^

Inflammatory markers are variably elevated at diagnosis: erythrocyte sedimentation rate (ESR) is increased in about 53%–55% and CRP in approximately 42%–56% of early RA patients, but both can be normal in up to 40% of cases.^30^, ^31^ This highlights the limited sensitivity of acute‐phase reactants and the need for integrating clinical, serological and imaging findings in early diagnostic assessment.

### 3.3 Imaging Features

Imaging modalities such as ultrasound (US) and magnetic resonance imaging (MRI) enhance early RA diagnosis and monitoring by detecting subclinical inflammation and structural changes not evident on clinical examination.^32^

**FIGURE 1:**
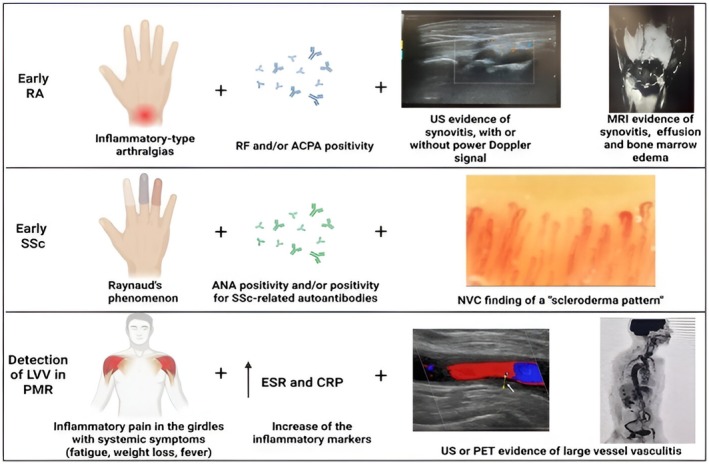
Combination of clinical features, laboratory, and imaging findings in early RA, SSc, and in the detection of LVV in patients with PMR. Illustration created with www.biorender.com and Microsoft PowerPoint. All images, except the vascular ultrasound in the lower panel, are original and belong to the Academic Division of Clinical Rheumatology, University of Genova, Italy. The vascular ultrasound image is reproduced from Schäfer VS. *Curr Rheumatol Rep*. 2023; 25 (9):279–289, licensed under a Creative Commons Attribution 4.0 International Licence (CC BY 4.0).^44^ CRP, C‐reactive protein; ESR, erythrocyte sedimentation rate; LVV, large vessel vasculitis; MRI, magnetic resonance imaging; PET, positron emission tomography; RA, rheumatoid arthritis; SSc, systemic sclerosis; US, ultrasound.

Compared to conventional radiography, US and MRI provide more sensitive information on inflammatory and soft tissue changes, including synovitis, joint effusion and early bone erosions (Figure 1). Baseline X‐rays of hands and feet are valuable for identifying pre‐existing structural damage and determining whether the disease is already erosive. However, their limited sensitivity in identifying active synovitis or early lesions makes US and in particular, MRI more suitable for capturing early inflammatory changes.^32^

#### 3.3.1 Applications of musculoskeletal ultrasound in early RA

Musculoskeletal ultrasound (US) plays a central role in early RA, improving diagnostic accuracy and supporting risk stratification in individuals with musculoskeletal symptoms. In seropositive patients without clinical arthritis, ultrasound abnormalities (grey scale synovitis, power Doppler signals, and erosions) can detect subclinical synovitis in 44%–51% of cases.

Notably, among ACPA‐positive individuals who later develop RA, approximately 80% have evidence of subclinical inflammation on imaging before the onset of inflammatory arthritis.^45^ A power Doppler score >2 (based on the semiquantitative OMERACT grading system) has been associated with an increased risk of progression to clinical arthritis, particularly in individuals who are double‐positive or at imminent risk of disease onset.^32^, ^33^, ^46^ Furthermore, recent studies have identified that the number of joints with subclinical inflammation is a key predictive factor, which, when combined with the presence of autoantibodies and an elevated ESR, is strongly associated with irreversible progression to RA.^47^

Recent studies underscore the critical role of musculoskeletal US in detecting subclinical inflammation and early structural changes among individuals at risk for RA, even before the clinical onset of overt synovitis. It has been demonstrated that power Doppler US not only detects subclinical synovitis but can also reveal bone erosions and loss of hyaline cartilage, which are critical markers of structural damage and predictors of persistent or progressive RA in patients with clinically suspect arthralgia. The presence of these US‐detected erosions, even in the absence of clinical arthritis, has been associated with a higher likelihood of disease progression, supporting US as a valuable tool for risk stratification and as an adjunct to serological and clinical assessment during the preclinical phase.^48^

Additionally, semiquantitative scoring systems, such as those developed by the OMERACT working groups, allow for standardized evaluation of both inflammation and early structural changes (cartilage loss, erosions) at sites most affected in early RA, particularly within the metacarpophalangeal joints. While bone erosions have historically been considered a late finding, emerging evidence confirms that US can detect subtle cortical breaks earlier than standard radiographs. Collectively, these findings emphasize the relevance and feasibility of US in both research and clinical practice for early detection, prognostication, and potential disease interception in preclinical RA.^49^

Additionally, the detection of tenosynovitis is becoming increasingly important in the assessment of patients with inflammatory joint pain.^50^ Compared to previous years, tenosynovitis is no longer considered merely an additional disease feature, but rather an early indicator that may even precede joint synovitis. Indeed, the identification of hand and foot tenosynovitis by MRI has been incorporated into the recent 2025 EULAR/ACR classification criteria for individuals with arthralgia at risk for RA.^51^

In patients with undifferentiated arthritis or equivocal clinical findings, US can confirm active synovitis and guide early therapeutic decisions. However, in the absence of clinical or serological inflammation, treatment should not be based solely on imaging findings to avoid overtreatment. Instead, US should be integrated with clinical and laboratory data, with a preference for close follow‐up and repeat imaging as needed.^52^

#### 3.3.2 Applications of MRI in early RA

MRI offers greater sensitivity than ultrasound in detecting early inflammatory changes, particularly bone marrow edema, a hallmark of imminent erosive progression that cannot be visualized by ultrasound.^53^ It is especially useful in cases of diagnostic uncertainty, when clinical and ultrasound findings are discordant or limited by technical factors (e.g. deep joints, obesity, or poorly accessible areas).^54^ MRI can also assist in differentiating RA from other inflammatory arthritides through detailed evaluation of synovitis and tenosynovitis.^31^

Of note, a recent observational study highlighted the need for cautious interpretation of MRI‐detected erosions in patients with clinically suspect arthralgia, as these findings did not correspond with radiographic erosive disease or disease progression, unless accompanied by subclinical inflammation, which showed a stronger association (OR 6.29 [95% CI 2.94–13.48]).^35^

It is also important to recognize that these inflammatory findings, such as synovitis and bone marrow edema, are not static and may regress, particularly with therapeutic intervention as seen in prevention trials (for more details, see Section 3.5).

Indeed, due to high cost, limited accessibility and longer acquisition times, MRI is not routinely used in clinical practice and remains a second‐line tool in early RA, particularly when imaging findings are expected to impact diagnostic or therapeutic decisions.

### 3.4 Predictive models for the future development of RA

In response to the need for targeted prevention and early intervention, several predictive models and risk stratification tools have been developed worldwide to estimate the likelihood that individuals with preclinical features or undifferentiated arthritis will progress to RA.

The Leiden Prediction Rule, developed in the Netherlands, is one of the most widely validated clinical prediction tools. This model integrates clinical parameters (such as joint involvement, symptom duration, morning stiffness), serological markers (ACPA, RF) and acute phase reactants to generate a weighted score corresponding to the probability of developing RA within 1 year. In both Western and non‐Western populations, a score ≥8 confers a high risk (approaching 100% in some cohorts), while a low score (<5) is associated with an excellent prognosis. The rule has shown strong predictive accuracy, with area under the curve (AUC) values ranging from 0.85 to 0.90.^55^

UK Risk Algorithms and other multivariate models such as the Swedish Epidemiological Investigation in RA have also been validated, incorporating demographic, clinical and serological data to predict RA in referred patients with early or undifferentiated arthritis. Some models also consider genetic risk scores or incorporate environmental factors such as smoking status.^56^

The EULAR/ACR 2025 Risk Stratification Criteria, previously mentioned, represent the latest consensus approach, derived from a collaborative analysis of 10 major international arthralgia cohorts (*n* > 2500 at‐risk subjects). This model provides a stepwise scoring system using clinical factors (morning stiffness, self‐reported joint swelling, difficulty making a fist), laboratory variables (CRP, RF, ACPA) and, optionally, MRI findings (especially tenosynovitis) to stratify patients by their likelihood of developing RA within 1 year. The inclusion of MRI‐detected subclinical inflammation further improves predictive value, with models achieving AUCs as high as 0.93. The criteria are designed to be applied flexibly, depending on the availability of imaging and serological resources, and support the selection of homogeneous risk groups for clinical trials or early intervention programs.^51^

### 3.5 Early intervention in rheumatoid arthritis

There is growing interest in strategies to delay or prevent clinical RA in high‐risk individuals, particularly those with arthralgia, seropositivity and imaging signs of subclinical inflammation. Several randomized trials have explored pharmacological interventions in this preclinical phase (Table 4).

The most compelling evidence for prevention comes from high‐quality randomized controlled trials (RCTs) targeting T‐cell co‐stimulation with abatacept. The APIPPRA and ARIAA trials showed that abatacept significantly reduced progression to RA, with a sustained benefit observed even 1 year after treatment discontinuation.^57^, ^58^ This supports the concept that early targeting of T‐cell co‐stimulation is a key preventive strategy.^59^ However, these trials also highlight a critical challenge, as a substantial proportion of individuals in the placebo arms (ranging from 43% to 63%) did not develop RA, raising concerns about potential overtreatment.

It is also important to contextualize these promising results. Longer‐term follow‐up from the ALTO study, the open‐label extension of APIPPRA, suggests the intervention primarily delays rather than permanently prevents RA onset. After treatment cessation, the cumulative incidence of arthritis in the original abatacept group began to converge with the placebo arm over the following year. Furthermore, the most significant and sustained benefit was concentrated in a hyper‐seropositive subgroup with a high antigenic burden, defined by positivity for five distinct autoantibody specificities (IgG ACPA, IgA ACPA, IgM RF, anti‐CarP, anti‐PAD antibodies).^60^ Since this specific antibody profile is not assessed in routine clinical practice, the broad applicability of this preventive strategy remains a challenge. These findings underscore the need for refined stratification tools to identify individuals most likely to achieve a durable benefit from early intervention.

Other strategies have yielded more limited effects, providing moderate‐level evidence. In the PRAIRI trial, the B‐cell depleting agent rituximab only temporarily delayed the onset of RA by approximately 12 months but failed to prevent it in the long term, as the cumulative incidence eventually converged with the placebo group.^61^

Similarly, the TREAT EARLIER study failed to meet its primary endpoint, as methotrexate plus glucocorticoids did not prevent the incidence of clinical arthritis. However, the treatment did lead to sustained improvements in symptoms, function and MRI inflammation, suggesting a role in modifying the disease burden rather than intercepting the disease itself.^62^

Finally, some approaches have provided low‐level evidence with no clear preventive benefit. The StopRA trial investigating hydroxychloroquine was stopped prematurely for futility after an interim analysis showed it was no better than placebo.^63^, ^64^ A trial by Bos et al. showed that a brief intervention with intramuscular dexamethasone in a small cohort produced only transient immunological effects without impacting the rate of arthritis development.^65^

Despite encouraging signals for targeted immunomodulation, particularly with abatacept, important challenges remain. The role of environmental risk factors, such as diet, smoking, certain beverages and even exogenous hormone use (e.g. oestrogens or hormone replacement therapy), should be carefully evaluated and if possible, correctly addressed early as part of preventive management.

Many at‐risk individuals will never develop RA, raising concerns about overtreatment. Moreover, predictive tools to identify those who would benefit most from early therapy are still suboptimal, and long‐term safety and cost‐effectiveness remain to be established.

Therefore, early intervention in seropositive individuals with subclinical inflammation shows promise, but further research is needed to refine patient selection and optimize combined therapeutic strategies in RA.

## 4 SYSTEMIC SCLEROSIS

Systemic sclerosis (SSc) is a rare and progressive autoimmune disease mainly affecting young to middle‐aged women, marked by immune dysfunction, microvascular damage, and progressive fibrosis of skin and internal organs.^66^ Despite its low worldwide prevalence (approximately 18 per 100,000 people), it has the highest mortality among rheumatic diseases, with prognosis depending on organ involvement.^67^

Clinically, SSc is classified into limited (lcSSc) and diffuse (dcSSc) cutaneous forms, which differ in severity and timing of organ complications^68^: dcSSc often leads to early and faster lung, heart or kidney involvement, while lcSSc progresses more slowly, with later pulmonary and gastrointestinal manifestations.^69^, ^70^

### 4.1 Clinical Features of early systemic sclerosis

Raynaud's phenomenon (RP) is often the earliest clinical manifestation of systemic sclerosis (SSc) and is characterized by episodic vasospasm of the digital arteries, typically triggered by cold or emotional stress. Clinically, it presents with a triphasic sequence of colour changes in the digits: pallor (ischemic phase) due to arterial vasoconstriction, followed by cyanosis (hypoxic phase) as deoxygenated blood pools in the venous system, and finally rubor (reperfusion phase) upon rewarming and restoration of blood flow.^71^

Although RP affects approximately 3%–5% of the general population, the vast majority of these cases are primary (idiopathic) and non‐progressive. However, a small proportion of patients (almost 14.6%) with RP develop secondary forms, most commonly in association with autoimmune connective tissue diseases such as SSc, and/or overlap connective tissue diseases (CTDs). In SSc, RP is not merely functional but reflects underlying structural microvascular abnormalities, including endothelial dysfunction, intimal vessel proliferation, obliterative vasculopathy and capillary loss, which explain its severity, chronicity and progression.^72^

To distinguish primary from secondary RP, imaging analysis is obtained with nailfold videocapillaroscopy (NVC) that is a fundamental non‐invasive tool. The identification of a characteristic ‘scleroderma pattern’ on NVC is a cornerstone of early diagnosis and a key component of the 2013 ACR/EULAR classification criteria for SSc (see Section 4.3 for a detailed description of imaging patterns).^73^, ^74^

The pivotal criteria for early systemic sclerosis (2001) by LeRoy and Medsger represent a valuable clinical framework for identifying patients in the initial stages of the disease, particularly among those presenting with RP.^7^ By emphasizing the combination of RP, SSc‐specific autoantibodies, and abnormal nailfold capillaroscopy findings, these criteria enable clinicians to recognize systemic sclerosis before overt skin or internal organ involvement emerges.

The subsequent study by Koenig et al. followed 586 patients with RP over a 20‐year period and demonstrated that the presence of both SSc‐specific autoantibodies (e.g. anti‐CENP‐B, anti‐topoisomerase I, anti‐RNAP III, anti‐Th/To) and a scleroderma‐type NVC pattern significantly increased the risk of progression to definite SSc (79.5% developed SSc; median time to progression 4.6 years; HR 60.08 versus patients with neither predictor).^75^ This study validated the study of LeRoy and Medsger.

Building upon the conceptual groundwork laid by LeRoy and Medsger, the Very Early Diagnosis of Systemic Sclerosis (VEDOSS) initiative, launched by the European Scleroderma Trials and Research (EUSTAR) group in 2011, further expanded the previous framework to enable very early identification of SSc in patients with RP.^76^

Unlike earlier criteria, VEDOSS introduced puffy fingers as a specific early clinical sign, often preceding skin fibrosis and associated with a higher risk of dcSSc.^77^, ^78^ However, the diagnostic utility of puffy fingers may vary in clinical practice, especially in ANA‐negative individuals. For example, in the EUSTAR‐VEDOSS cohort, puffy fingers were reported only in 38.5% of ANA‐positive patients but only 23.3% of ANA‐negative patients with Raynaud's.^79^

Moreover, puffy fingers are not exclusive to SSc and may also appear in overlap syndromes such as undifferentiated connective tissue disease (UCTD) or mixed connective tissue disease (MCTD), for the latter disease being recognized as early and diagnostic clinical manifestations in validated criteria.^80^ This underscores the need for careful differential diagnosis in very early presentations.

Recent data from two large U.S. cohorts, GENISOS and CONQUER, challenge the idea that RP is the most common initial symptom of SSc. Between 31% and 44% of patients first showed non‐RP signs like puffy fingers or hand swelling, often linked to diffuse cutaneous SSc, joint contractures and RNA Polymerase III antibodies. This pattern was especially frequent in Black patients, where RP; however, can be harder to detect.^81^, ^82^

These results highlight the need to recognize non‐RP presentations as an early disease form in some populations (see Table 2). However, without systematic microvascular assessments such as NVC, early microvasculopathy may be missed and at least early diagnosis compromised; see 2013 ACR/EULAR classification criteria for SSc.^73^, ^74^ Genetic, environmental, and healthcare differences also limit applying these findings to other populations.

**TABLE 2:** Clinical and laboratory elements for the early diagnosis of SSc and baseline assessment for organ involvement.

	Finding	Main notes
Elements for early diagnosis of SSc		
Clinical features	Raynaud's phenomenon (RP)	Often the earliest clinical manifestation, present in over 95% of patients^83^
	Puffy fingers	A specific early clinical sign, however present in only 18%–39% of patients in VEDOSS cohorts^84^
	Skin sclerosis	Skin thickening, the hallmark of SSc, is present in 75% of patients and can begin early, especially in the diffuse cutaneous subset.^83^
	Digital ulcers	These painful sores on the fingertips or toes can be an early sign of more severe vascular disease, occurring in 34% of patients.^85^
	Musculoskeletal symptoms	Arthralgias (joint pain) are quite common, affecting 40%–60% of patients. Frank arthritis (joint inflammation) can also be seen in up to 16% of cases.^86^
	Gastrointestinal symptoms	Issues like reflux, difficulty swallowing, and bloating are very common, affecting up to 71% of patients, and can be an early indicator of internal organ involvement.^85^
	Dyspnea	Shortness of breath is reported by 20%–50% of patients and is a critical symptom that may signal the presence of early interstitial lung disease (ILD) or pulmonary arterial hypertension (PAH).^85^
Laboratory markers	Autoantibodies	Antinuclear antibodies (ANA): A key screening tool, positive in >90% of SSc patients^87^
		SSc‐specific Autoantibodies^87^
		ACA positive in 20%–35% of SSc patients: Associated with limited cutaneous SSc (lcSSc) and risk of pulmonary arterial hypertension (PAH). Sensitivity 29%–31%, Specificity 87%–97%
		Anti‐Scl‐70 positive in 30%–40% of patients: linked to diffuse cutaneous SSc (dcSSc), frequent digital ulcers, and high risk of severe interstitial lung disease (ILD). Sensitivity 33%–58%, Specificity 95%–99%
		Anti‐RNAPol3 positive in 10%–20% of patients: defines subset with rapidly progressive skin fibrosis, renal crisis, gastric vascular lesions, cancer risk near onset; ILD uncommon. Sensitivity 7%–13%, Specificity 97%–100%
		Anti‐Th/To positive in 2%–5% of patients: associated to lcSSc with high risk of ILD and PAH; associated with shorter lag time between RP and SSc onset128. Sensitivity 2%–4%, Specificity 98%–99%
		Anti‐U3‐RNP (fibrillarin) positive in 2%–8% of patients: frequently seen in Afro‐Caribbean men, associated with early age at onset, PAH, and GI involvement129 Sensitivity 1%–7%, Specificity 97%–100%
		Anti‐PM/Scl positive in 3%–10%: typically seen in SSc–myositis overlap, associated with ILD, calcinosis, dermatomyositis‐like rash, and favourable ILD outcomes in the first decade130. Sensitivity 5%–11%, specificity 93%–98%
		Anti‐Ku positive in 2%–7% of patients: hallmark of PM/SSc overlap syndrome, associated with myositis, ILD, and low prevalence of vascular complications131. Sensitivity 3%–5% and Specificity 96%–98%
Imaging	Nailfold videocapillaroscopy (NVC)	The gold standard for assessing SSc‐related microangiopathy. The ‘scleroderma pattern’ is classified into ‘Early’, ‘Active’ and ‘Late’ stages according to disease progression^88^ New pre‐scleroderma ‘very early’ NVC changes recognized^89^
Baseline assessment for organ involvement		
Imaging and function: Lungs	High‐resolution CT (HRCT)	Mandatory at diagnosis to screen for interstitial lung disease (ILD), even in asymptomatic patients; detects subclinical ILD in ~40%–50% of cases.^90^
	Pulmonary function tests (PFTs)	Should be performed at baseline and serially for monitoring, but are not sensitive enough for initial screening alone^90^
Imaging: Heart	Transthoracic echocardiography (TTE)	The first‐line screening tool for pulmonary arterial hypertension (PAH) and cardiac involvement at diagnosis.^91^
	Cardiac MRI	Used in selected cases for a more detailed assessment of myocardial fibrosis or inflammation when echocardiography is inconclusive^92^
Imaging: Skin and peripheral microvasculature	High‐frequency skin US	Can detect early dermal changes (thickness, stiffness); remains a promising research tool requiring further validation.^93^
	Laser speckle contrast analysis (LASCA)	A research tool for assessing skin perfusion that correlates with NVC findings; not yet standardized for clinical use^94^
Other assessments	Gastrointestinal and renal screening	Tests such as oesophageal manometry or renal ultrasound are not performed routinely at baseline and are reserved for symptomatic patients or when there is clinical suspicion of involvement^95^, ^96^

Abbreviations: ACA, anti‐centromere antibodies; ANA, antinuclear antibodies; Anti‐Ku, anti‐Ku antibodies; Anti‐PM/Scl, anti‐PM/Scl antibodies; Anti‐RNAPol3, anti‐RNA polymerase III antibodies; Anti‐Scl‐70, anti‐topoisomerase I antibodies; Anti‐Th/To, anti‐Th/To antibodies; Anti‐U3‐RNP, anti‐U3‐ribonucleoprotein (fibrillarin) antibodies; dcSSc, diffuse cutaneous systemic sclerosis; GI, gastrointestinal; HRCT, high‐resolution computed tomography; ILD, interstitial lung disease; LASCA, laser speckle contrast analysis; lcSSc, limited cutaneous systemic sclerosis; MRI, magnetic resonance imaging; NVC, nailfold videocapillaroscopy; PAH, pulmonary arterial hypertension; PFTs, pulmonary function tests; PM/SSc, polymyositis/systemic sclerosis overlap; RP, raynaud's phenomenon; SSc, systemic sclerosis; TTE, transthoracic echocardiography; US, ultrasound; VEDOSS, very early diagnosis of systemic sclerosis.

Recent NVC studies are starting to identify ‘very early’ pre‐scleroderma microvascular changes (‘very early’ and progressive capillary dilations up to giant validated capillaries) in SSc patients with RP, which may offer further insight.^89^

### 4.2 Laboratory Features

Serological evaluations are pivotal in the identification of early SSc, particularly among patients initially presenting with RP. ANA are present in over 90% of SSc patients and represent a key screening tool. However, a subset of patients with ANA‐negative SSc has also been described, often characterized by less vasculopathy but more severe gastrointestinal involvement and worse prognosis.^97^

Among disease‐specific autoantibodies, the most widely recognized and clinically useful are anti‐centromere (ACA), anti‐topoisomerase I (anti‐Scl70), and anti‐RNA polymerase III (anti‐RNAPol3).^98^ Not only do these antibodies aid the diagnosis, but they also enable clinical and prognostic stratification.

ACA is typically associated with the limited cutaneous subset (lcSSc) and a higher risk for future pulmonary arterial hypertension (PAH).^99^, ^100^

Anti‐Scl‐70 antibodies are highly specific for SSc and are linked to the diffuse cutaneous subset (dcSSc), with an increased risk of severe interstitial lung disease (ILD).^69^

Anti‐RNAPol3 antibodies also mark a diffuse cutaneous subset, often with rapid skin progression and a significant risk for developing scleroderma renal crisis.^101^

In addition to these classical markers, novel SSc‐related autoantibodies have been described such as anti‐eIF2B, anti‐RuvBL1/2, anti‐U11/U12 RNP (RNPC3) and anti‐BICD2 in up to 10% of so‐called ‘seronegative’ patients, often associated with aggressive or overlapping disease phenotypes, including ILD, myopathy, severe GI or myocardial involvement and, in the case of RNPC3 and eIF2B, possible paraneoplastic syndromes.^87^

The accurate detection of these autoantibodies requires appropriate methods. Indirect immunofluorescence (IIF) on HEp‐2 cells remains the standard for ANA screening, but many SSc‐specific antibodies require more refined techniques such as immunoblotting, multiplex line immunoassays, or immunoprecipitation (the latter more rarely applied) to be reliably identified.^87^

### 4.3 Imaging findings in early SSc

Imaging of the microvasculature and internal organs plays a central role in the early identification and monitoring of SSc. Among the morphological tools assessing peripheral microcirculation, NVC remains the gold standard for evaluating structural microvascular abnormalities in patients with RP^102^ (Figure 1).

The typical ‘scleroderma pattern’ observed in SSc has been classified and standardized by Cutolo et al. in 2000 into ‘early’, ‘active’ and ‘late’ stages following the pathophysiological evolution of SSc microangiopathy.^88^ The ‘early’ pattern is marked by a few giant capillaries and mild haemorrhages with preserved density; the ‘active’ pattern includes frequent haemorrhages and moderate capillary loss; and the ‘late’ pattern is defined by severe capillary loss, disorganized architecture and neoangiogenesis.^103^, ^104^ This structured classification allows both diagnostic and prognostic use of NVC in early SSc.

More recently, the detection of a capillary with a diameter >30 μm and <50 μm (at least two capillaries per linear mm at NVC) in patients with Raynaud's phenomenon, before the development of a full‐blown scleroderma pattern, has been proposed as a ‘pre‐scleroderma signature’, indicative of a ‘very early’ step of SSc.^89^ However, this finding is under further validation in prospective cohorts before it can be fully integrated into routine clinical assessment.

In addition to the morphological assessment, laser speckle contrast analysis (LASCA) offers a dynamic and non‐invasive method to evaluate peripheral blood perfusion. It provides high‐resolution, contact‐free imaging of skin microcirculation, with perfusion indices that correlate inversely with the severity of NVC abnormalities.^94^, ^105^ Currently, LASCA remains a research tool, and further studies are required to establish its clinical utility, reproducibility, and standardization across SSc cohorts.^106^ A more recent development in SSc vascular imaging is the DAVIX (Digital Artery Volume Index), an MRI‐based technique that quantifies digital artery volume as a marker of vascular fibrosis. Recent studies have shown that DAVIX can predict the future development of digital ulcers and correlates with pulmonary arterial hypertension risk and overall disease severity, positioning it as a promising new outcome measure for vascular involvement in SSc.^107^

### 4.4 Baseline screening for internal organ involvement

Skin involvement in SSc is traditionally assessed using the modified Rodnan skin score (mRSS) at 17 sites of the arms and trunk, but advanced imaging techniques like high‐frequency ultrasound and MRI are being investigated for more objective evaluation. High‐frequency skin ultrasound can detect early changes not visible on clinical exam and allows for quantifying dermal thickness, echogenicity and stiffness using elastography.^108^, ^109^, ^110^

While international guidelines support standardized protocols, skin ultrasound remains primarily a research tool pending further validation and standardization before widespread clinical use.^93^

High‐resolution computed tomography (HRCT) of the chest is recommended for the screening of ILD in all patients at the time of SSc diagnosis, regardless of symptoms. This is supported by the ACR and the American College of Chest Physicians, as well as by international consensus, due to the high prevalence and prognostic significance of subclinical ILD in early SSc detectable in 41%–50% of patients depending on the stratification of disease subset.^111^ Recent advancements in SSc imaging include the promising use of lung ultrasound (LUS) as a screening tool for ILD. Multiple studies demonstrate that LUS, by detecting B‐lines, can identify subclinical and established ILD with a high sensitivity and, crucially, a high negative predictive value (up to 100%), particularly in low‐risk ILD patients when compared to HRCT.^112^ This suggests that LUS may offer a practical, radiation‐free alternative to HRCT in excluding ILD, especially for initial screening or in settings where HRCT is impractical. However, major and further studies are needed to optimize the real specificity, standardize protocols and mainly to determine the best timing for LUS during disease monitoring.^113^

Pulmonary function tests (PFTs), including spirometry, lung volumes and DLCO, should also be performed at baseline and serially for monitoring, but PFTs alone are insufficient for initial screening because they may miss early or subclinical ILD.^90^

Transthoracic echocardiography is recommended at diagnosis to screen for pulmonary arterial hypertension (PAH) and cardiac involvement.^114^ Recently, however, cardiac MRI has gained increasing relevance in selected cases, offering a more detailed assessment of myocardial fibrosis, inflammation and function, particularly when echocardiographic findings are inconclusive or clinical suspicion of cardiac involvement persists.^92^

Barium swallow and oesophageal manometry are not recommended as routine screening tests in asymptomatic patients with early SSc; these studies should be reserved for patients who have symptoms of dysphagia, gastroesophageal reflux, or when there is clinical suspicion of gastrointestinal involvement, such as unexplained pulmonary symptoms and signs, including chronic cough, recurrent aspiration pneumonia, voice hoarseness or radiographic evidence of oesophageal dilation.^95^

Similarly, renal imaging (e.g. renal ultrasound) is not routinely recommended for screening in early SSc unless there are clinical or laboratory signs suggestive of scleroderma renal crisis or other renal pathology.^96^

Altogether, multimodal and multiorgan imaging in early SSc is necessary to promptly detect organ involvement, optimize risk stratification and guide early intervention.

### 4.5 Early Intervention in early systemic sclerosis

The rationale for early therapeutic intervention in SSc lies in the recognition that tissue fibrosis is preceded and perpetuated by early immune dysregulation and endothelial damage.^66^ While clinical data support early immunosuppressive treatment in patients with dcSSc or ILD, evidence of benefit in the very earliest stages, defined by Raynaud's phenomenon, SSc‐specific autoantibodies and abnormal NVC, remains limited and inconclusive.

Canadian registry data have shown an increased use of immunosuppressants, especially methotrexate and MMF since 2007, guided mainly by autoantibody profiles rather than skin severity.^115^

Supporting this approach, low‐level evidence from a single‐center retrospective study by Yomono et al. found that starting immunosuppressants within 18 months of SSc diagnosis reduced lung function decline, disease activity and improved event‐free survival^116^ (see more details in Table 4). Similarly, moderate‐level evidence from the prospective observational European Scleroderma Observational Study (ESOS) showed that methotrexate, MMF and cyclophosphamide improved skin thickness in early diffuse SSc, and untreated patients had worse survival^117^; however, the differences between treatment groups were not statistically significant, and the observational design is susceptible to confounding by indication.

Limited data in limited cutaneous SSc suggest early immunosuppression may reduce long‐term organ damage, but evidence on preventing lung disease is inconsistent. These findings underscore the need for controlled trials and tailored treatments, especially due to low biologic use and possible confounding factors.^118^

In this context, the ‘Hit Hard and Early’ trial, a small RCT, provided evidence against untargeted immunosuppression in the very earliest disease stages. This study explored whether prompt intervention in the earliest clinical phases of SSc, specifically in patients with puffy fingers and autoantibody positivity, could alter disease trajectory.^119^ However, very early initial high‐dose glucocorticoids alone did not improve capillary density, disease progression, or lung function at 1 year, discouraging their use in the absence of overt inflammatory organ involvement.

In addition to traditional immunosuppressants, antifibrotic therapies such as nintedanib have become valuable for treating SSc‐associated ILD, supported by high‐level evidence from a large RCT. Subgroup analyses from the SENSCIS trial showed that patients with early disease (<18 months from first non‐Raynaud symptom) experienced faster FVC decline (−167.8 mL/year vs. −93.3 mL/year in the overall population) and appeared to benefit more from nintedanib, highlighting the potential advantage of early antifibrotic intervention in rapidly progressive cases.^120^

As for vasodilators, calcium channel blockers, phosphodiesterase‐5 (PDE‐5) inhibitors, and endothelin receptor antagonists, they are effective for managing symptoms of Raynaud's phenomenon and digital ulcers, with bosentan reducing new ulcer formation.^121^ However, no data supports their role as disease‐modifying treatments in early or pre‐clinical SSc stages. A large French study found that only sildenafil may have cardioprotective effects in established SSc, improving diastolic function and ejection fraction, while bosentan, ACE inhibitors and iloprost showed no significant cardiac benefits.^122^

In summary, while early intervention in SSc is a compelling strategy, especially for patients at high risk of progression (i.e. male sex, anti‐Scl70 positivity and black ethnicity), the overall quality of evidence for broad immunosuppressive therapy remains low to moderate, largely derived from observational or retrospective studies that are prone to bias.^123^ The risk of overtreatment, including adverse effects from immunosuppressive or biologic agents, must be balanced against the uncertain benefit in preclinical disease stages.

Future research should prioritize longitudinal cohort studies and targeted randomized trials in early or at‐risk SSc populations, to better define when, who and how to treat and to move from symptom control to true disease modification.

## 5 EARLY DETECTION OF GIANT CELL ARTERITIS IN PATIENTS WITH POLYMYALGIA RHEUMATICA

Polymyalgia rheumatica (PMR) is quite a common inflammatory disease in elderly people with a peak of incidence at 70–75 years of age, causing pain and stiffness in the shoulders, neck and hips.^124^ It can occur alone or alongside giant cell arteritis (GCA), a vasculitis affecting large arteries, especially the temporal arteries and the aorta.^8^ These conditions are now seen as part of a shared spectrum (GCA‐PMR spectrum disease) due to overlapping mechanisms and treatment responses.^8^, ^125^

Clinical or imaging findings of GCA are detected in 16%–23% of patients initially diagnosed with PMR.^126^ However, the precise pathological meaning and optimal management for this subclinical vascular inflammation remain subjects of ongoing debate. Early recognition of large vessel vasculitis among PMR patients is crucial, given the potential for severe vascular complications such as vision loss, strokes and aortic aneurysms if left untreated.^127^

### 5.1 Clinical features

The clinical presentation of GCA in patients with PMR is heterogeneous. While some patients present with classic cranial symptoms such as new‐onset headache, jaw claudication or visual changes, many others have underlying subclinical GCA without these overt signs, posing a significant diagnostic challenge.^127^ In studies of GCA cohorts, PMR symptoms co‐occurred with cranial involvement in about 42% of patients (Table 3).^135^

**TABLE 3:** Features for the detection of subclinical large‐vessel vasculitis (LVV) in patients with polymyalgia rheumatica (PMR).

	Finding	Main notes
Clinical and laboratory features suggesting underlying LVV in PMR patients		
Clinical and laboratory features suggesting underlying LVV	Constitutional symptoms	Persistent fever, unexplained weight loss, and significant fatigue are present in 30%–50% of cases.^126^, ^128^
	Atypical or refractory PMR	Polymyalgic symptoms that are resistant to standard doses of glucocorticoids (e.g. 12.5–15 mg of prednisone) may indicate underlying vascular involvement^129^
	Vascular symptoms	Limb claudication, particularly in the upper extremities, can be seen in 10%–20% of patients with extracranial GCA^130^
Laboratory markers	Acute phase reactants	ESR and CRP are elevated in over 90% of patients, though they have limited specificity.^128^
	Other markers	Increased concentrations of angiopoietin‐2/1 ratios and matrix MMP‐3 might potentially identify GCA in PMR patients but not yet validated for routine clinical use^131^

	Diagnostic role	Prevalence of findings
Imaging modalities for detecting LVV in patients presenting with a polymyalgic syndrome		
Imaging: musculoskeletal ultrasound (US)	US of the temporal and axillary arteries is recommended as the first‐line imaging modality to detect mural inflammatory changes in patients with suspected GCA, with an axillary artery intima‐media thickness of ≥1.0 mm demonstrating high specificity for large vessel vasculitis^132^, ^133^	Detects subclinical GCA in approximately 23% of steroid‐naïve PMR patients who lack cranial symptoms.^134^
Imaging: PET/CT	Offers sensitive, whole‐body imaging for detecting inflammation in large vessels, especially extracranial arteries	Finds evidence of LVV (vascular uptake ≥ liver) in 12%–29% of PMR cases, with higher rates in those with persistent symptoms^134^

Abbreviations: CRP, C‐reactive protein; ESR, erythrocyte sedimentation rate; GCA, giant cell arteritis; LVV, large‐vessel vasculitis; MMP‐3, matrix metalloproteinase‐3; PET/CT, positron emission tomography/computed tomography; PMR, polymyalgia rheumatica; US, ultrasound.

However, these symptoms may be absent in many PMR patients who nonetheless harbor underlying subclinical GCA, posing significant diagnostic challenges.^136^ Indeed, a subset of patients initially presenting with isolated PMR shows subclinical large‐vessel GCA, involving extracranial arteries such as the aorta and its primary branches, including carotid, subclavian and axillary arteries.

The clinical features of extracranial involvement may be non‐specific, contributing to a diagnostic delay. These patients frequently experience systemic or constitutional symptoms, such as persistent fever of unknown origin, unexplained weight loss, night sweats, significant fatigue or malaise.^126^ Additionally, polymyalgic symptoms refractory to standard doses of glucocorticoids (typically 12.5–15 mg of prednisone equivalent daily sufficient in isolated PMR) may suggest underlying vascular involvement, warranting further investigation for GCA.^129^

Recent studies have also highlighted that the occurrence of limb claudication and pulse discrepancies between limbs can be early clinical markers of extracranial GCA, although these findings are often subtle and may go unnoticed without careful clinical examination.^137^ Furthermore, patients with subclinical large‐vessel GCA may present with isolated elevation of inflammatory markers without clear focal symptoms, which can lead to misinterpretation as isolated PMR.^126^, ^136^

Given these varied clinical presentations, recent guidelines recommend systematic assessment of potential GCA symptoms (cranial and extracranial) and routine physical examination (palpation of temporal arteries, assessment of peripheral pulses, blood pressure measurements in both arms) in all patients initially diagnosed with PMR, particularly those resistant to standard therapy or presenting with persistent systemic symptoms.^127^, ^136^, ^137^

### 5.2 Laboratory findings

Laboratory markers in GCA and PMR commonly include elevated acute phase reactants such as ESR and CRP, which, although useful for disease activity monitoring, have limited specificity for discriminating isolated PMR from associated GCA.^128^

A recent study has identified that a CRP cut‐off value of ⩾26.5 mg/L in PMR might help the identification of subclinical GCA, but these results warrant further validation in other cohorts.^138^ Specifically, an ESR >60 mm/h (positive LR 2.40, 95% CI 1.71–3.36) and CRP ≥2.5 mg/dL (negative LR 0.38, 95% CI 0.25–0.59) are informative, but neither marker alone is diagnostic, and both can be elevated in isolated PMR or other inflammatory conditions.^134^

Elevated platelet count (>400 × 10^3^/μL) is also associated with GCA and may aid in risk stratification. Recent biomarker studies have highlighted the potential of angiopoietin‐2 and matrix metalloproteinase‐3 (MMP‐3) as promising indicators of underlying GCA in PMR patients.^131^ These biomarkers, although preliminary, demonstrate higher specificity for vascular inflammation than traditional acute phase reactants and, despite being currently used in research settings, might be used in the future also in clinical practice.^139^

### 5.3 Imaging features of large vessel vasculitis in PMR patients

Imaging plays a central role in the early detection of subclinical GCA in patients with PMR. When large vessel vasculitis such as GCA is suspected in a patient, recent EULAR recommendations endorse Colour Doppler ultrasound (US) as the first‐line imaging modality. This is due to its high sensitivity and specificity for cranial GCA and its ability to detect the characteristic halo sign indicating vessel wall edema. The assessment of both temporal and axillary arteries is specifically recommended, as axillary involvement is common and contributes to diagnostic yield and disease monitoring. This reflects a conceptual shift toward viewing GCA as a single disease spectrum with overlapping cranial and extracranial manifestations.^10^

Recent large prospective and multicentre studies have shown that up to 23%–29% of patients with newly diagnosed PMR and no cranial symptoms display vascular abnormalities on imaging, particularly involving extracranial arteries such as the axillary, subclavian, and carotid branches.^134^ These abnormalities include non‐compressible arteries and increased intima‐media thickness or halo signs, more frequently detected in extracranial than cranial districts, indicating a predominant large‐vessel pattern of subclinical inflammation in this patient population.^140^

In patients presenting with overt GCA and visual symptoms, transorbital ultrasound has also emerged as a valuable adjunct. This technique can detect early biomarkers of ocular involvement, such as reduced blood flow in the central retinal artery, which helps identify patients at increased risk for vision loss.^141^

Positron emission tomography/computed tomography (PET/CT) offers whole‐body imaging and is particularly sensitive for detecting large‐vessel inflammation, especially in extracranial arteries such as the axillary, subclavian, and carotid arteries, which may be missed by US. However, cost, availability, and radiation exposure limit its use in routine screening.

Meta‐analyses and large prospective studies have shown that up to 23%–29% of newly diagnosed PMR patients without cranial symptoms have subclinical GCA detectable by imaging (Figure 1). Current evidence suggests that these patients may be at a higher risk of relapse and progression to overt GCA.^134^, ^142^ However, cost, availability and radiation exposure limit its routine use.

Several clinical and laboratory features have been proposed as predictive factors for the development of GCA in PMR patients. These include older age at onset, constitutional symptoms, hip or pelvic girdle pain (possibly reflecting limb claudication), lower back pain, night sweats, resistance to initial glucocorticoid treatment, and markedly elevated acute phase reactants.^126^ Due to the relatively frequent coexistence of subclinical GCA in PMR, routine imaging screening in PMR patients at high risk (persistent symptoms, elevated inflammatory markers despite therapy, older age at disease onset) is increasingly suggested.^127^

### 5.4 Early intervention and clinical outcomes of PMR patients with large vessel vasculitis

Timely initiation of glucocorticoid therapy remains the cornerstone in managing GCA, but the optimal treatment approach for subclinical large‐vessel vasculitis (LVV) in PMR remains uncertain. Current expert recommendations suggest starting glucocorticoids in PMR patients with clear imaging evidence of LVV, even in the absence of classic GCA symptoms, to reduce the risk of complications.^137^ However, no standardized therapeutic protocol exists for these cases, and randomized trials are lacking.

Moderate‐level evidence from a prospective, multicenter ultrasound study by De Miguel et al. that imaging‐detected LVV may identify a more aggressive disease phenotype, associated with higher relapse rates (62% vs. 16%, *p* < 0.001), greater glucocorticoid burden, and increased need for immunosuppressants like methotrexate.^129^ In particular, rapid tapering of glucocorticoids appears to increase relapse risk in this subgroup. However, these observational findings must be interpreted with caution, as clinicians were not blinded to the ultrasound results, which influenced treatment decisions. Nevertheless, the prognostic significance of subclinical vasculitis remains debated. While US‐based studies report increased relapse risk, others using FDG‐PET do not confirm this, likely due to methodological differences (see Table 4 for more details). At the moment, current evidence is heterogeneous and largely observational, with a high risk of bias from confounding by indication, warranting cautious interpretation and individualized management strategies.^149^ Notably, the absence of systematic vascular assessment in most PMR trials may have contributed to underestimating the true clinical impact of subclinical vasculitis in this setting.^150^

**TABLE 4:** Evidence from randomized controlled trials and/or observational studies in treating earlier RA and SSc and outcomes of subclinical or clinically manifest large vessel vasculitis in PMR.

Trial (intervention)	Population included	Intervention vs. Placebo	Primary outcome	Key results	Follow‐up	Level of evidence/key limitations
RA						
APIPPRA^57^	213 ACPA+ and RF+ patients with inflammatory joint pain	Abatacept 125 mg weekly subcutaneous for 12 months vs. placebo	Time to clinical synovitis in ≥3 joints or RA (2010 criteria)	6% vs. 29% developed arthritis at 12 months; 25% vs. 37% at 24 months. Sustained improvements in pain, function, and quality of life	24 months	High (RCT): Strong evidence for prevention with sustained benefit, but the risk of overtreating individuals who would not progress is a key concern
ARIAA^58^	98 ACPA+ patients with arthralgia and inflammation at MRI	Abatacept 125 mg weekly subcutaneous for 6 months vs. placebo	Proportion with improved MRI inflammation at 6 months	57% vs. 31% showed MRI improvement; 8% vs. 35% developed RA during treatment (HR 0.14)	18 months	High (RCT): Reduced MRI inflammation and RA progression in a high‐risk group, but the smaller sample size and use of a surrogate primary endpoint are limitations
PRAIRI^61^	81 ACPA+ and RF+ patients with arthralgia	Single infusion of rituximab 1000 mg vs. placebo	Time to development of clinical arthritis	55% risk reduction at 12 months (HR 0.45), 12‐month delay in arthritis development, but no sustained effect	29 months mean	Moderate (RCT): The effect was temporary, suggesting the intervention postponed but did not permanently alter the disease course
TREAT EARLIER^143^	236 patients with arthralgia and MRI subclinical inflammation	Single glucocorticoid injection + methotrexate up to 25 mg/week for 1 year vs. placebo	Development of clinical arthritis (2010 RA criteria or ≥2 joints) persisting ≥2 weeks	No prevention of arthritis (19% vs. 18%, HR 0.81), but sustained improvement in pain, function, and MRI inflammation	2 years	Moderate (RCT): Did not meet its primary prevention endpoint, but provides good evidence for modifying the disease burden rather than intercepting the disease
StopRA Trial^63^	142 anti‐CCP3+ individuals	Hydroxychloroquine 200‐400 mg daily vs. placebo for 1 year	Development of inflammatory arthritis classified as RA or IA with ≥1 erosion	No difference: 34% HCQ vs. 36% placebo developed RA at 3 years (*p* = 0.844)	3 years	Low (RCT‐Halted): Findings are from an interim analysis that showed a clear lack of benefit, leading to the trial's termination
Bos et al^65^	83 ACPA+/RF+ patients with arthralgia	Two intramuscular dexamethasone injections (100 mg each) vs. placebo	50% reduction or normalization of autoantibody levels at 6 months	ACPA reduced 22% and IgM‐RF 14% vs. placebo, but no difference in arthritis development (20% vs. 21%)	26 months median	Low (RCT): A small trial with a very brief intervention that showed no impact on the clinical outcome of arthritis development.

Study and design	Population included	Intervention vs. Placebo	Primary outcome	Key results	Follow‐up	Level of evidence/key limitations
SSC						
SENSCIS^120^ (randomized controlled trial)	SSc‐ILD patients; subgroup analysis of early disease (<18 months since first non‐Raynaud symptom)	Nintedanib vs. placebo	Rate of FVC decline over 52 weeks	Nintedanib reduced FVC decline more in early disease subgroup (−167.8 mL/year decline in placebo)	1 year	High (RCT‐subgroup): Strong evidence from a large RCT that nintedanib reduces FVC decline, particularly in early SSc‐ILD. Limitations are that this is a subgroup analysis and the benefit was specific to ILD, not overall disease.
Hit hard and early^119^ (randomized controlled trial)	Very early disease (puffy fingers <3 years, VEDOSS criteria)	Methylprednisolone 1000 mg IV vs. placebo	Nailfold capillary density at 12 weeks	No significant difference in capillary density (−0.5, 95% CI −1.1 to 0.2) or secondary outcomes (including disease progression, lung function, patient‐reported outcomes)	1 year	Low to moderate (RCT‐Negative): A small proof‐of‐concept RCT that failed to show any benefit of high‐dose glucocorticoids in very early SSc, suggesting this specific approach is ineffective.
ESOS^117^ (Prospective observational)	Early dcSSc (within 3 years of skin thickening onset, median 11.9 months)	MTX, MMF, cyclophosphamide vs. no immunosuppressant	Change in modified Rodnan skin score	mRSS change at 12 months: MTX −4.0, MMF −4.1, CYC −3.3, No IS −2.2 (*p* = 0.346). Survival at 24 months: MTX 94.1%, MMF 88.8%, CYC 90.1%, No IS 84.0% (*p* = 0.440)	2 years	Low to moderate (prospective observational): A large observational study suggesting a weak, non‐significant trend favouring immunosuppressants. The non‐randomized design makes it prone to confounding by indication.
Yomono et al^116^ (single centre retrospective cohort)	Early vs delayed intervention comparison (≤18 months vs. >18 months at treatment start)	Early vs. delayed intervention with CYC, MMF, MTX, tocilizumab	Clinical worsening events	Early intervention superior: lower clinical worsening (revised ACR‐CRISS *p* = 0.03, PF‐ILD *p* = 0.003)	1 year	Low (retrospective cohort): Supports a ‘window of opportunity’ but has a high risk of bias due to its retrospective, single‐center design and small sample size (*n* = 46).
Guedon et al^122^ (prospective observational study)	Mixed early/established disease (79 patients, 13.7%, had <18 months since first non‐Raynaud symptom)	Sildenafil, bosentan, ACE inhibitors, iloprost vs. no treatment	Heart‐related outcomes (diastolic dysfunction, EF <50%, PAH)	Only sildenafil showed benefit: −2.83% diastolic dysfunction, −0.88% altered EF	3 years	Low to moderate (prospective observational): Large cohort study providing exploratory, hypothesis‐generating data for sildenafil's potential cardioprotective effect. Findings are limited by the observational design.

Study	Detection method	Prevalence of large vessel vasculitis	Impact on outcomes	Treatment changes	Follow‐up	Level of evidence/key limitations
Subclinical GCA in PMR						
De Miguel et al^129^	Ultrasound	23% (50/150 patients)	4× Higher Relapse Rate: 62% vs. 16% (p < 0.001) PMR with subclinical GCA: 31/50 (62%) relapsed Isolated PMR: 16/100 (16%) relapsed	Higher starting prednisone doses (36.6 vs. 19.8 mg daily, *p* < 0.001) Faster tapering = higher relapse risk	22 months	Moderate (prospective cohort): Evidence that ultrasound‐detected subclinical GCA is linked to a higher relapse risk. Limited by the non‐randomized, unblinded design.
Lavado‐Pérez et al^144^	18F‐FDG PET/CT	65% (26/40 patients with LVV suspicion)	73% Had good clinical/laboratory response: 23% had poor response despite treatment	85% Treatment changes: 17/20 previously treated patients had therapy modifications 6 patients: increased steroids 11 patients: increased steroids + added MTX/tocilizumab	NA	Low to moderate (observational): Demonstrates the clinical utility of PET/CT in guiding therapy, but provides limited data on patient outcomes (e.g. relapse rates).
Arévalo Ruales et al^145^	18F‐FDG PET/CT	48% (11/23 patients)	Higher CRP levels in LVV group (40.26 vs. 22.29 mg/L, p = 0.03)	80% of LVV patients required DMARDs vs. 25% in isolated PMR More biological therapy use (28% vs. 2%)	NA	Low (retrospective): The high prevalence of LVV is likely due to significant selection bias, as only patients with an atypical or poor‐response course were scanned. Small sample size (*n* = 23).
Moreel et al^146^	18F‐FDG PET/CT	9% (31/337 patients)	Higher glucocorticoid doses needed throughout follow‐up No difference in treatment duration or relapse rate	Higher GC starting dose and doses during follow‐up until 12 months	26 months	Low to moderate (retrospective cohort): Provides important conflicting evidence, suggesting PET findings may not predict relapse. Limited by retrospective design and confounding by treatment.
Emamifar et al.^147^	18F‐FDG PET/CT	71.4% had PMR activity, 2.6% had GCA activity	Treatment response independent of PET/CT findings. Several predictors of poor response identified	No difference in treatment based on imaging findings	40 weeks	Low (prospective cohort): This study focused on clinical predictors, not imaging outcomes. It found that initial PET/CT findings did not predict clinical treatment response, providing low‐quality evidence for the prognostic value of PET imaging.
Blockmans et al^148^	18F‐FDG PET/CT	31% had vascular uptake	58% relapse rate overall. No correlation between PET findings and relapse risk	PET results did not predict treatment response or relapse	37.0 ± 11.4 months	Low to moderate (prospective): An early prospective study that questions the prognostic value of PET scans for relapse. Limited by small size and older PET technology.

Abbreviations: ACE, angiotensin‐converting enzyme; ACPA, anti‐citrullinated peptide antibodies; CRISS, Composite Response Index in diffuse cutaneous Systemic Sclerosis; CYC, cyclophosphamide; DMARDs, disease‐modifying antirheumatic drugs; EF, ejection fraction; FVC, forced vital capacity; GC, glucocorticoid; GCA, giant cell arteritis; HCQ, hydroxychloroquine; HR, hazard ratio; HRCT, high‐resolution computed tomography; IA, inflammatory arthritis; ILD, interstitial lung disease; IS, immunosuppressant; IV, intravenous; LVV, large vessel vasculitis; MMF, mycophenolate mofetil; MRI, magnetic resonance imaging; MTX, methotrexate; PAH, pulmonary arterial hypertension; PET/CT, positron emission tomography/computed tomography; PF‐ILD, progressive fibrosing interstitial lung disease; PMR, polymyalgia rheumatica; RA, rheumatoid arthritis; RCT, randomized controlled trial; RF, rheumatoid factor; SSc, systemic sclerosis; TTE, transthoracic echocardiography; VEDOSS, very early diagnosis of systemic sclerosis.

The emerging concept of the combined GPSD entity supports the idea that patients with PMR and vascular involvement represent a biologically distinct and more aggressive phenotype, rather than a simple coexistence of two entities.^151^, ^152^

Despite these important insights, optimal therapeutic approaches remain incompletely defined for patients with PMR‐associated subclinical large‐vessel involvement. While glucocorticoid dosing regimens for clinically overt GCA typically involve at least twice the dosage recommended for isolated PMR, observational data from the De Miguel cohort suggest that PMR patients with subclinical GCA often receive intermediate doses (e.g. 20–30 mg prednisone/day), but relapse despite these regimens, indicating that a ‘PMR‐style’ approach may be insufficient.^129^

Long‐term vascular outcomes such as aneurysms or stenosis are poorly characterized, and medium‐term studies have not demonstrated significant ischemic progression, though follow‐up data remain limited.^153^ Given these uncertainties, routine vascular imaging may be considered for selected high‐risk PMR patients, particularly those with persistent symptoms, poor response to therapy, or elevated inflammatory markers, while broader screening strategies will require the development of clinical stratification tools.

Ultimately, randomized controlled trials stratifying patients based on vascular imaging findings are needed to determine whether tailored treatment improves outcomes and to define evidence‐based management strategies for this increasingly recognized subset of PMR.

## 6 LIMITATIONS OF THE CURRENT EVIDENCE BASE

While this narrative review aims to provide a comprehensive and balanced synthesis of the current literature, several potential sources of bias should be acknowledged. Firstly, publication bias, favouring studies with positive or novel findings, remains an inherent limitation, particularly as evidence for early diagnosis and intervention in rheumatic diseases is often shaped by high‐profile trials or guidelines rather than negative or null results. In addition, the review integrates studies with differing designs, populations, and definitions of ‘early’ disease, resulting in substantial heterogeneity that may limit direct comparability between the considered conditions. The predominance of European and North American cohorts, variable classification criteria, and evolving technologies (e.g. imaging or biomarker assays) further contribute to this heterogeneity.

Most of the included studies were appraised qualitatively, and thus conclusions may reflect subjective interpretation of study robustness and clinical applicability. Moreover, evidence gaps remain in some areas, particularly for preclinical phases and early intervention in SSc and LVV/PMR, where randomized controlled trials are scarce and observational or expert opinion predominates. As such, recommendations and interpretations should be considered within the context of these methodological limitations, and there is a need for larger, diverse and prospective studies to strengthen the evidence base and reduce the risk of selection, reporting and publication biases in this field.

## 7 CONCLUSION

The landscape of early diagnosis and intervention in IARDs has evolved toward proactive identification strategies, though with markedly different levels of evidence across conditions. Our overview shows that while early detection capabilities have advanced significantly through integrated clinical, serological, and imaging approaches, the translation to effective interventions varies considerably.

Evidence for early intervention is most robust in RA, especially in ACPA positive patients, where abatacept has demonstrated significant benefits in preventing disease progression in seropositive individuals with subclinical inflammation. However, important challenges remain in identifying which at‐risk individuals will actually develop disease, raising concerns about potential overtreatment.

In SSc, while laboratory and imaging advances, in particular thanks to the progress in identifying the earliest microvascular damage (i.e. NVC) at the earliest step of the disease, therapeutic interventions, especially early immunosuppressive therapies in preclinical disease stages, remain largely not yet standardized and are under progressive investigation.

For PMR, the recognition of subclinical LVV as a distinct phenotype has important prognostic implications, yet optimal management strategies remain undefined due to conflicting evidence from different imaging modalities and study designs.

Moving forward, more nuanced approaches are required to balance early intervention benefits against risks of overtreatment. Priority areas include developing better predictive algorithms to identify patients most likely to progress, conducting adequately powered randomized trials in truly and well‐defined early disease stages and establishing cost‐effective screening strategies.

## AUTHOR CONTRIBUTIONS

**Elvis Hysa**: Conceptualization; methodology; investigation; data curation; writing—original draft preparation; writing—review and editing; resources; visualization. **Emanuele Gotelli**: Conceptualization; investigation; writing—original draft preparation; writing—review and editing; visualization. **Carmen Pizzorni**: Investigation; data curation; writing—review and editing; visualization. **Sabrina Paolino**: Investigation; data curation; writing—review and editing. **Alberto Sulli**: Methodology; investigation; writing—review and editing; supervision. **Vanessa Smith**: Methodology; investigation; writing—review and editing; supervision. **Rosanna Campitiello**: Conceptualization; methodology; supervision; writing—review and editing; resources. **Maurizio Cutolo**: Conceptualization; methodology; investigation; project administration and draft finalization; supervision; writing—review and editing; resources; visualization; final approval.

## FUNDING INFORMATION

The authors have not declared a specific grant for this research from any funding agency in the public, commercial or not‐for‐profit sectors.

## CONFLICT OF INTEREST STATEMENT

The authors declare no competing interests.

## Data Availability

This narrative review is based on published literature. All data supporting the conclusions are available in the cited references. No new datasets were generated during this study.
